# Benefits and challenges of Big Data in healthcare: an overview of the European initiatives

**DOI:** 10.1093/eurpub/ckz168

**Published:** 2019-11-18

**Authors:** Roberta Pastorino, Corrado De Vito, Giuseppe Migliara, Katrin Glocker, Ilona Binenbaum, Walter Ricciardi, Stefania Boccia

**Affiliations:** 1 Sezione di Igiene, Istituto di Sanità Pubblica, Università Cattolica del Sacro Cuore, Rome, Italy; 2 Department of Public Health and Infectious Diseases, Sapienza University of Rome, Rome, Italy; 3 Division of Medical Informatics for Translational Oncology, German Cancer Research Center, Heidelberg, Germany; 4 Department of Biology, University of Patras, Patras, Greece; 5 Department of Woman and Child Health and Public Health—Public Health Area, Fondazione Policlinico Universitario A. Gemelli IRCCS, Rome, Italy

## Abstract

Healthcare systems around the world are facing incredible challenges due to the ageing population and the related disability, and the increasing use of technologies and citizen’s expectations. Improving health outcomes while containing costs acts as a stumbling block. In this context, Big Data can help healthcare providers meet these goals in unprecedented ways. The potential of Big Data in healthcare relies on the ability to detect patterns and to turn high volumes of data into actionable knowledge for precision medicine and decision makers. In several contexts, the use of Big Data in healthcare is already offering solutions for the improvement of patient care and the generation of value in healthcare organizations. This approach requires, however, that all the relevant stakeholders collaborate and adapt the design and performance of their systems. They must build the technological infrastructure to house and converge the massive volume of healthcare data, and to invest in the human capital to guide citizens into this new frontier of human health and well-being. The present work reports an overview of best practice initiatives in Europe related to Big Data analytics in public health and oncology sectors, aimed to generate new knowledge, improve clinical care and streamline public health surveillance.

## Introduction

Data have become an omnipresent concept in our daily lives with the routine collection, storage, processing and analysis of immense amount of data. This characteristic is cross-sectorial, ranging from the domain of machine learning and engineering, to economics and medicine.

Over the last decades, there has been growing enthusiasm of the potential usefulness of these massive quantities of data, called Big Data, in transforming personal care, clinical care and public health.^1^

### An overview of Big Data definitions

Despite the term Big Data having become ubiquitous, there is no universal definition until now on the use of this term. According to McKinsey the term Big Data refers to *datasets whose size is beyond the ability of typical database software tools to capture, store, manage, and analyse*.^2^ Gartner proposed the popular definition of Big Data with the ‘3V’: *Big Data is volume, high-velocity and high-variety information assets that demand cost-effective, innovative forms of information processing for enhanced insight and decision making*.^3^ According to other definitions, instead, Big Data is also characterized by a fourth dimension: *Veracity, concerning the quality, authenticity, ‘trustworthiness’ of data.*^4^

Furthermore, there is an emergent discussion that ‘Big’ is no longer the defining parameter, but rather how ‘smart’ the data are, focusing on the insights that the volume of data can reasonably provide.^5^ This aspect is fundamental in the health sector. The potential of Big Data in improving health is enormous. However, its potential value is unlocked only when leveraged to drive decision making and, to enable such evidence-based decision making, it is necessary to have efficient processes to analyse and turn high volumes of data into meaningful insights.

A specific definition of what Big Data means for health research was proposed by the Health Directorate of the Directorate-General for Research and Innovation of the European Commission: *Big Data in health encompasses high volume, high diversity biological, clinical, environmental, and lifestyle information collected from single individuals to large cohorts, in relation to their health and wellness status, at one or several time points*.^6^

### Big Data analytics for health systems

**Figure 1:**
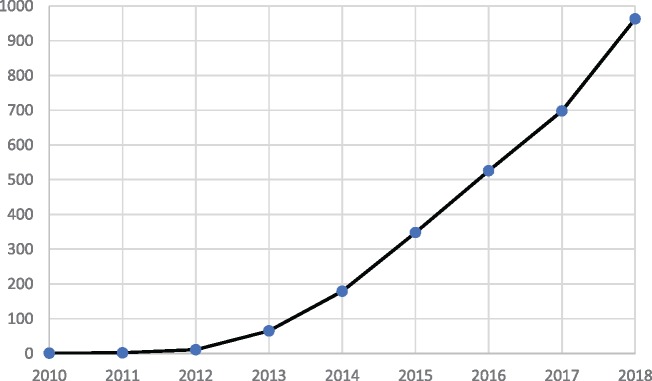
Number of publications on ‘Big Data and health’ reported by year (from 2010 to 2018). The publications are identified through a search of MEDLINE with the following terms for the literature search: (‘Big Data’) AND (‘Health’)

The complexity of Big Data analysis arises from combining different types of information, which are electronically captured. The last years have seen an explosion of new platforms, tools and methodologies in storing, and structuring such data, followed by a growth of publications on Big Data and Health (figure 1). To date, we can collect data from electronic healthcare records, social media, patient summaries, genomic and pharmaceutical data, clinical trials, telemedicine, mobile apps, sensors and information on well-being, behaviour and socio-economic indicators.

Healthcare professionals can, therefore, benefit from an incredibly large amount of data. Recent reports suggest that US healthcare system alone stored around a total of 150 exabytes of data in 2011 with the perspective to reach the yottabyte.^7^

Starting with the collection of individual data elements and moving to the fusion of heterogeneous data coming from different sources, can reveal entirely new approaches to improve health by providing insights into the causes and outcomes of disease, better drug targets for precision medicine, and enhanced disease prediction and prevention.

In light of this, opportunities and potential are enormous for the benefit of patients and, in general, of the healthcare system.

### The potential benefits of Big Data for healthcare in the European Union

Big Data is a sensitive issue for European Union (EU) institutions: the availability of health-related Big Data can have a positive impact on medical and healthcare functions. EU is faced with several changes that may affect the sustainability of its healthcare system. By 2025 life expectancy is expected to further increase, and this may result in more people living longer, but not necessarily in a healthy and active condition. This will put further pressure on Europe’s healthcare costs and economic productivity.

In this context, the data sharing approach can improve outcomes for patients and evidence-based healthcare decision making as reported during the workshop on ‘Digitalisation and Big Data: implications for the health sector’, held on 19 June 2018 at the European Parliament.^8^

The use of Big Data in healthcare, in fact, can contribute at different levels as reported by the Study on Big Data in Public Health, Telemedicine and Healthcare of the European Commission:^9^ (i) *increasing earlier diagnosis and the effectiveness and quality of treatments* by the discovery of early signals and disease intervention, reduced probability of adverse reactions, etc. (sector 1); (ii) *widening possibilities for prevention of diseases* by identification of risk factors for disease (sector 2); (iii) *improvement of pharmacovigilance and patient safety* through the ability to make more informed medical decisions based on directly delivered information to the patients (sector 3); (iv) *prediction of outcomes* (sector 4).

In the next paragraphs, examples of EU initiatives in the four macro sectors are listed.

Big Data have the potential to yield new insights into risk factors that lead to disease. There is the possibility to engage with the individual patient more closely and import data from mobile health applications or connected devices. These data have the potential to be analysed and used in real-time to prompt changes in behaviours that can reduce health risks, reduce harmful environmental exposures or optimize health outcomes.

Finally, Big Data can help identify and promptly intervene on high-risk and high-cost patients.^10^ Effective ways of managing these data can therefore facilitate precision medicine by enabling detection of heterogeneity in patient responses to treatments and tailoring of healthcare to the specific needs of individuals.^11^ All these aspects should eventually lead to a reduction in inefficiency and improvement in cost containment for the healthcare system.

Examples of Big Data analytics for new knowledge generation, improved clinical care and streamlined public health surveillance are already available. Below we report a selection of best practices in Europe in the public health and oncology fields.

### Big Data in public health

Efforts to improve the availability and accessibility of data in the EU appear to be driven mainly by socio-economic purposes. However, great importance is placed on the need of using data and new information and communication technology (ICT) in public health to improve quality of prevention and care.

In the next years, European health systems must respond more efficiently to the exponential increase of chronic patients identifying the most efficient interventions and releasing the full potential of ICT. The e-health platforms that many European governments are trying to implement can be effective in improving the management of chronic patients in the community setting by interfacing between different health professionals and specialists and with the patients. Furthermore, a large part of EU citizens uses the internet looking for information on health and access to health services.

Moreover, Big Data and predictive analytics can contribute to precision public health by improving public health surveillance and assessment, therefore, in a public health perspective, the gathering of a very large amount of data, constitute an inestimable resource to be used in epidemiological research, analysis of the health needs of the population, evaluation of population-based intervention and informed policy making.^9^

Many projects across the EU are exploring the potential of available Big Data in a wide range of fields. A systematic review published in 2016 from the European Commission identified at that time 10 priority projects on Big Data implemented in Europe that fall in the four macro sectors described above and are aimed to support the sustainability of health systems by addressing the improvement of the quality and effectiveness of treatment, fighting chronic disease and supporting healthy lifestyles.^9^ Some of these projects focussed on gathering a very wide range of data types, from GP records, hospitalizations, drug prescription and laboratory and radiology analyses in order to create comprehensive national data warehouses. Among them, the ‘Decision Support for Health Policy and Planning: Methods, Models and Technologies based on Existing Health Care Data’ (DEXHELPP), the eHealth project in Estonia, the ARNO observatory in Italy and the Hospital Episode Statistics in the United Kingdom. The DEXHELPP project (mainly regarding sectors 1 and 4) used routinely collected health data sources to analyse the performance of the health system, to forecast future changes and to simulate the application of policy and interventions. The Estonian eHealth project (mainly regarding sectors 1, 2 and 3) was more oriented toward the improvement of the quality and efficiency of health services, aiming to digitalize all the information and prescription of each patient. Furthermore, in order to facilitate data collection, they provide an environment called X-Road to which all healthcare providers can link while using their own ICT solutions. The ARNO project (mainly regarding sector 4), was committed to epidemiological research, giving the possibility of deep stratification of the general population. The Hospital Episode Statistics (mainly regarding sector 4) was in charge of the Secondary Uses Service that publishes reports and analyses to support the National Health Service in the delivery of healthcare services.

Beside these projects characterized by a comprehensive approach, other initiatives focused on specific conditions (e.g. chronic conditions, rare diseases and psychiatric disorders) are also available in the EU.^9^ Among them, the PASSI surveillance project (mainly regarding sectors 1 and 2) in Italy provides large amount of information on the life style of almost the 90% of the population, enabling to individuate specific targets to implement and assess public health actions. The Shared Care Platform (mainly regarding sectors 1 and 3) in Denmark is focused on chronic patients, aiming to harmonize the course of treatment among health and social care providers. The Spanish Rare Diseases Registries Research Network (SpainRDR) (mainly regarding sector 1) focuses on the development of clinical research on rare diseases, providing the harmonization and unification into one comprehensive platform of pre-existing databases and registries of rare diseases. CEPHOS-LINK (mainly regarding sectors 1, 2 and 4), is a platform dedicated to mental health that involves six EU countries. It is committed to collect data on psychiatric hospital admissions and re-admissions, with the aim of finding determinants of re-admissions and to harmonize the psychiatric care pathways across the EU.

In addition to the projects reported above, the EU’s framework programmes for research and innovation funded a large number of initiatives on Big Data in public health. In table 1, we list 11 projects funded from the EU between 2012 and 2018 with a contribution over €499.999 that are captured from the Cordis website (source: cordis.europa.eu).

**Table 1:** EU supported initiatives concerning activities that involve the use of Big Data in public health in Europe from 2012 to 2018, in chronological order (EU contribution from: 499.999€)

Project acronym	Full title	Coordinator country	Start date	End date
FATE	Fall detector for the elderly	Spain	01/03/12	31/05/15
MIRRI	Microbial resource research infrastructure	Germany	01/11/12	30/04/16
EXPOSOMICS	Enhanced exposure assessment and omic profiling for high priority environmental exposures in Europe	UK	01/11/12	30/04/17
ADMOS	Advertising monitoring system development for outdoor media analytics	Hungary	01/09/13	31/08/15
DRIVE-AB	Driving Re-investment in R&D and responsible antibiotic use	Sweden	01/10/14	31/12/17
MARIO	Managing active and healthy aging with use of caring service robots	Ireland	01/02/15	31/01/18
METASPACE	Bioinformatics for spatial metabolomics	Germany	01/07/15	30/04/18
ComPat	Computing patterns for high performance multiscale computing	Netherlands	01/10/15	30/09/18
City4Age	Elderly-friendly city services for active and healthy ageing	Italy	01/12/15	30/11/18
i-PROGNOSIS	Intelligent Parkinson eaRly detectiOn Guiding NOvel Supportive InterventionS	Greece	01/02/16	31/01/20
ROADMAP	Real world outcomes across the AD spectrum for better care: multimodal data Access Platform	UK	01/11/16	31/10/18

*Source*: CORDIS, https://cordis.europa.eu/en, retrieved on 05.07.2019.

*Query*: contenttype=‘project’ AND exploitationDomain/code=‘health’ AND (‘public’ AND ‘health’ AND ‘data’ AND ‘“big’ AND ‘data”’) AND/project/ecMaxContribution>=499999.

*Notes*: Four projects (iManagerCancer, MedBionformatics, Mocha, Iasis) that involve the use of Big Data in oncology (table 2) result also from the query above.

### Big Data in oncology

Cancer is one of the major health problems affecting our society, a situation that is set to deteriorate globally as the population grows and ages. According to the State of Health in the EU reports, cancer is recognized as one of the major contributors to premature deaths in the EU. It also has an impact on the economy in terms of lower labour market participation and productivity. Advances in Big Data analytics are given cancer researchers powerful new ways to extract value from diverse sources of data.

These diverse sources include a huge amount of data for one patient. As cancer is a molecularly highly complex disease with an enormous intra- and intertumoral heterogeneity among different cancer types and even patients, the collection of various different types of omics data can provide a unique molecular profile for each patient and significantly aid oncologists in their effort for personalized therapy approaches.^12^

The approach of combining these sources of data is implemented in Comprehensive Cancer Centres (CCCs).^13^ One of 13 CCCs in Germany is the National Center of Tumor Diseases, where the Molecularly Aided Stratification for Tumor Eradication Research (MASTER) trial is conducted (mainly regarding sector 1, 2 and 3). In a recent review article,^14^ this trial was illustrated as an example of a highly successful programme addressing the molecular profiling in cancer patients. Within the MASTER trial data relevant to diagnostic information of young patients with advanced-stage cancer diseases is collected by performance of whole exome or whole genome sequencing and RNA sequencing, analysed and discussed.

Another example for a success story given in the review is the INdividualized therapy FOr Relapsed Malignancies in children (INFORM) (mainly regarding sector 1, 2 and 3) registry which aims to address relapses of high-risk tumours in paediatric patients. Data from whole-exome, low-coverage whole-genome, RNA sequencing and microarray-based DNA methylation profiling are utilized to identify patient-specific therapeutic targets. The INFORM registry started as a national effort in Germany and has been extended with the participation of eight European countries, as well as Australia.

Next to the described projects there are many other initiatives which focus on the value of Big Data in oncology, the EU alone funds more than 90 projects working on this topic (projects with a funding over €499.999 are listed in table 2). A potential umbrella for bringing together national efforts such as those mentioned above at the European level is Cancer Core Europe.^15^

**Table 2:** EU supported initiatives concerning activities that involve the use of Big Data in oncology in Europe, in chronological order (EU contribution from: 499.999€)

Project acronym	Full title	Coordinator country	Start date	End date
ClouDx-i	Cloud based software solution for next generation diagnostics in infectious diseases	Ireland	07/01/13	06/01/17
iManageCancer^a^	Empowering patients and strengthening self-management in cancer diseases	Germany	01/02/15	31/07/18
MedBioinformatics^a^	Creating medically-driven integrative bioinformatics applications focused on oncology, CNS disorders and their comorbidities	Spain	01/05/15	30/04/18
MOCHA^a^	Models of child health appraised	United Kingdom	01/06/15	30/11/18
ENLIGHT-TEN	European network linking informatics and genomics of helper T cells	Germany	01/10/15	30/09/19
PERMIDES	Personalized medicine innovation through digital enterprise solutions	Germany	01/09/16	31/08/18
IASIS^a^	Integration and analysis of heterogeneous Big Data for precision medicine and suggested treatments for different types of patients	Greece	01/04/17	31/05/20

*Source*: CORDIS, https://cordis.europa.eu/en, retrieved on 05.07.2019.

*Query*: contenttype=‘project’ AND exploitationDomain/code=‘health’ AND ((‘tumor’ OR ‘tumour’ OR ‘cancer’ OR ‘oncology’) AND (‘“big data”’)) AND/project/ecMaxContribution>=499999.

a Initiatives on Big Data and oncology arising also from the public health query.

Collaborations are of extremely high importance especially in the case of paediatric or other rare types of cancer, where the data collected for one patient is indeed enormous, however the number of patients a single centre can have access to is too low to obtain statistical power high enough to reach meaningful results. One of the main challenges of these collaborations is the access to the data as well as the opportunity to analyse the huge amount of data in an efficient way. Physicians, researchers and informatics experts can only benefit from collected data and expert knowledge when they get easy and intuitive access to own data or data of partners. For example, at the German Cancer Research Center, tools are developed to grant ways to access and analyse own data together with data from partners.

Additionally, international exemplary approaches of sharing data among partners or the public are done by The Cancer Genome Atlas (TCGA) and the International Cancer Genome Consortium (ICGC) which provide researchers with access to thousands of sequenced patients with different types of cancer. The availability of these data jointly with data by other partners has enabled large meta-analyses and machine learning algorithms, integrating different types of cancer that led to the identification of novel cancer driver genes that belong to specific pathways and can be possible therapy targets. Furthermore, there are a number of public databases that provide access to catalogues of mutations found to be involved in cancer, such as the Catalogue of Somatic Mutations in Cancer (COSMIC).

All these multiple sources of information combined and the establishment and support of CCCs across Europe offer the potential to increase the number of patients that can be offered molecular profiling and individualized treatment based on Big Data analysis.

### Ethical and legal issues for the effective use of Big Data in healthcare

The use of Big Data in healthcare poses new ethical and legal challenges because of the personal nature of the information enclosed.

Ethical and legal challenges include the risk to compromise privacy, personal autonomy, as well as effects on public demand for transparency, trust and fairness while using Big Data.^16^

Data heterogeneity, data protection, analytical flows in analysing data and the lack of appropriate infrastructures for data storage emerged as critical technical and infrastructural issues that might endanger a Big-Data-driven healthcare.

Recently, Skovgaard et al.^17^ explored attitudes among people living in the EU toward the reuse of health data. The review indicates that the use of health data for purposes other than treatment enjoys support among people, as long as the data are expected to further the common good. In this context, the recent call reported in *Science* from a number of eminent scientists worldwide, for the unrestricted use of public genomic data, finds a fertile ground from the public.^18^ Concerns evolve around the commercialization of data, data security and the use of data against the interests of the people providing the data.

The recent EU Data Protection Regulation (GDPR) tries to balance patients’ privacy while ensuring patient’s data can be shared for healthcare and research purposes.

On 23 January 2017 the Consultative Committee of the Council of Europe’s data protection convention adopted ‘Guidelines on the protection of individuals with regard to the processing of personal data in a world of Big Data’,^19^ the first document on privacy and Big Data which provides suggested measures for preventing any possible negative effects of the use of Big Data on human rights and freedoms.

Therefore, any government that uses Big Data in the health sector needs to establish affirmative policies to protect the health data of individuals, in terms of confidentiality, privacy and security, while ensuring that advancements in science can take advantage from the open use of data for the community well-being.

## Conclusions

Big Data is beginning to revolutionize healthcare in Europe as it offers paths and solutions to improve health of individual persons as well as to improve the performance and outcomes of healthcare systems.

The implementation of precision medicine remains contingent on significant data acquisition and timely analysis to determine the most appropriate basis on which to tailor health optimization for individual prevention, diagnosis and disease treatment. Achieving effective and proportionate governance of health-related data will be essential for the future healthcare systems, and it requires that stakeholders collaborate and adapt the design and performance of their systems to reach the maximum innovative potential of information and innovation technology on health in the EU.

In this context, EU Member States should agree on international technical standards, taking also into account openness that is considered as the basic paradigm for digital transformation. Additionally, new approaches must be found for translating the vast amount of data into meaningful information that healthcare professionals can use. Further efforts must be made to make information for doctors and health professionals more accessible and understandable.

To achieve this, existing training and education programmes for healthcare professionals should integrate the issues of data handling in the curricula to ensure the development of the necessary skills and competencies. This is one of the objectives of the ‘European network staff eXchange for integrAting precision health in the health Care sysTems’ consortium (ExACT)^20^ project that aims to integrate precision health in European health systems by training a new generation of healthcare professionals across and outside of the EU.

In conclusion, we are living in fast-moving times, not least in terms of healthcare innovation. Whilst there are pressing needs for more personalized and sustainable health services, science and technology are offering a host of potentially invaluable new tools to deliver them. A cooperation at the EU level is needed to facilitate investments both in new technology and in the human capital, in order to guide citizens into this new frontier of human health and well-being where data are becoming a significant corporate asset, a vital economic input and the foundation of new business models.

*Conflicts of interest*: None declared.

## References

[1] Vayena E, Dzenowagis J, Brownstein JS, Sheikh A Policy implications of big data in the health sector. Bull World Health Organ 2018;96:66–8. 29403102 10.2471/BLT.17.197426 PMC5791870

[2] Manyika J, Chui M, Brown B, et al Big data: The next frontier for innovation, competition, and productivity. 2011 www.mckinsey.com/mgi (12 September 2019, date last accessed).

[3] Beyer M, Laney D The Importance of ‘Big Data’: A Definition. Gartner report. 2012, 1–9.

[4] McAfee A, Brynjolfsson E Big data: the management revolution. Harv Bus Rev 2012;90:60–66, 68, 128. 23074865

[5] George G, Haas MR, Pentland A Big Data and management. AMJ 2014;57:321–6.

[6] Auffray C, Balling R, Barroso I, et al Making sense of big data in health research: towards an EU action plan. Genome Med 2016;8:71. 27338147 10.1186/s13073-016-0323-y PMC4919856

[7] Raghupathi W, Raghupathi V Big data analytics in healthcare: promise and potential. Health Inf Sci Syst 2014;2:3. 25825667 10.1186/2047-2501-2-3 PMC4341817

[8] Boccia S, Pastorino R, Giraldi L Digitalisation and Big Data: implications for the health sector, Policy Department for Economic, Scientific and Quality of Life Policies. Available at: http://www.europarl.europa.eu/RegData/etudes/IDAN/2018/619030/IPOL_IDA(2018)619030_EN.pdf (12 September 2019, date last accessed).

[9] European Commission. Study on Big Data in public health, telemedine and healthcare. 2016 Available at: https://ec.europa.eu/health/sites/health/files/ehealth/docs/bigdata_report_en.pdf%0A%0A (12 September 2019, date last accessed).

[10] Bates DW, Saria S, Ohno-Machado L, et al Big Data in health care: using analytics to identify and manage high-risk and high-cost patients. Health Aff 2014;33:1123–31. 10.1377/hlthaff.2014.0041 25006137

[11] Hopp WJ, Li J, Wang G Big Data and the precision medicine revolution. Prod Oper Manag 2018;27:1647–64.

[12] Jameson JL, Longo DL Precision medicine—personalized, problematic, and promising. N Engl J Med 2015;372:2229–34. 26014593 10.1056/NEJMsb1503104

[13] van Harten WH Comprehensive cancer centres based on a network: the OECI point of view. Ecancermedicalscience 2014;8:ed43. 25228921 10.3332/ecancer.2014.ed43 PMC4162677

[14] Joos S, Nettelbeck DM, Reil-Held A, et al German Cancer Consortium (DKTK) - a national consortium for translational cancer research. Mol Oncol 2019;13:535–42. 30561127 10.1002/1878-0261.12430 PMC6396349

[15] Eggermont AMM, Apolone G, Baumann M, et al Cancer core Europe: a translational research infrastructure for a European mission on cancer. *Mol Oncol* 2019;13:521–7. 10.1002/1878-0261.12447 PMC6396377 30657633

[16] Ienca M, Ferretti A, Hurst S, et al Considerations for ethics review of big data health research: a scoping review. PLoS One 2018;13:e0204937. 30308031 10.1371/journal.pone.0204937 PMC6181558

[17] Skovgaard LL, Wadmann S, Hoeyer K A review of attitudes towards the reuse of health data among people in the European Union: the primacy of purpose and the common good. Health Policy 2019;123:564–71. 30961905 10.1016/j.healthpol.2019.03.012 PMC6558994

[18] Amann RI, Baichoo S, Blencowe BJ, et al Toward unrestricted use of public genomic data. Science 2019;363:350–2. 30679363 10.1126/science.aaw1280

[19] Guidelines on the protection of individuals with regard to the processing of personal data in a world of Big Data Consultative Committee of the Convention for the Protection of Individuals with Regard to Automatic Processing of Personal Data (T-PD) Guidelines 1 on the Protection of Individuals with Regard to the Processing of Personal Data in a World of Big Data 2 Directorate General of Human Rights and Rule of Law. www.coe.int/data-protection (12 September 2019, date last accessed).

[20] Boccia S, Pastorino R, Mariani M, Ricciardi W The European network staff eXchange for integrAting precision health in the health Care sysTems (ExACT): a Marie Curie Research and Innovation Staff Exchange (RISE) project. Epidemiol Biostat Public Heal 2019;16. doi: 10.2427/13122.

